# Rational Design and Evaluation of an Artificial *Escherichia coli* K1 Protein Vaccine Candidate Based on the Structure of OmpA

**DOI:** 10.3389/fcimb.2018.00172

**Published:** 2018-05-23

**Authors:** Hao Gu, Yaling Liao, Jin Zhang, Ying Wang, Zhiyong Liu, Ping Cheng, Xingyong Wang, Quanming Zou, Jiang Gu

**Affiliations:** ^1^National Engineering Research Center of Immunological Products, Department of Microbiology and Biochemical Pharmacy, College of Pharmacy, Third Military Medical University, Chongqing, China; ^2^Department of Critical Care Medicine, Children's Hospital of Chongqing Medical University, Chongqing, China; ^3^Department of Laboratory Medicine, Southwest Hospital, Third Military Medical University, Chongqing, China

**Keywords:** structure-based vaccine design, meningitis, *Escherichia coli* K1, outer membrane protein A, extracellular loops

## Abstract

*Escherichia coli* (*E. coli*) K1 causes meningitis and remains an unsolved problem in neonates, despite the application of antibiotics and supportive care. The cross-reactivity of bacterial capsular polysaccharides with human antigens hinders their application as vaccine candidates. Thus, protein antigens could be an alternative strategy for the development of an *E. coli* K1 vaccine. Outer membrane protein A (OmpA) of *E. coli* K1 is a potential vaccine candidate because of its predominant contribution to bacterial pathogenesis and sub-cellular localization. However, little progress has been made regarding the use of OmpA for this purpose due to difficulties in OmpA production. In the present study, we first investigated the immunogenicity of the four extracellular loops of OmpA. Using the structure of OmpA, we rationally designed and successfully generated the artificial protein OmpAVac, composed of connected loops from OmpA. Recombinant OmpAVac was successfully produced in *E. coli* BL21 and behaved as a soluble homogenous monomer in the aqueous phase. Vaccination with OmpAVac induced Th1, Th2, and Th17 immune responses and conferred effective protection in mice. In addition, OmpAVac-specific antibodies were able to mediate opsonophagocytosis and inhibit bacterial invasion, thereby conferring prophylactic protection in *E. coli* K1-challenged adult mice and neonatal mice. These results suggest that OmpAVac could be a good vaccine candidate for the control of *E. coli* K1 infection and provide an additional example of structure-based vaccine design.

## Introduction

*Escherichia coli* (*E. coli*) K1 is a Gram-negative bacterium that commonly causes meningitis in neonates (Scheld et al., 2002). This bacterium initially colonizes nasopharyngeal or gastrointestinal sites. After penetration into the blood circulation, where it multiplies, *E. coli* K1 invades human brain microvascular endothelial cells (HBMECs) and causes damage to brain tissues (Xie et al., 2004). Despite the conventional application of antibiotics and supportive care, the morbidity, and mortality rates of *E. coli* K1-associated neonatal meningitis remain unchanged (Nau et al., 2015; van de Beek et al., 2016). The fatality rates of *E. coli* K1-infected infants range from 5 to 30%, and the survivors often exhibit life-time sequelae, such as mental retardation, cortical blindness, and hearing loss (Croxen and Finlay, 2010; van de Beek et al., 2010). Therefore, an effective vaccine is urgently needed for the effective control of *E. coli* K1 infection.

Traditionally, capsular polysaccharides (CPs) have been considered good candidates for vaccine development because of their contribution to bacterial virulence and sub-cellular localization. The core antigens of many successful vaccines come from CPs, such as vaccines against *Haemophilus influenzae* type b (Zarei et al., 2016), *Pneumococci* (Geno et al., 2015), and *Salmonella typhi* (Cavallari and De Libero, 2017). However, O-acetylated colominic acid (CA) produced by *E. coli* K1 is considered a self-antigen due to its similarity to polysaccharides found on the surface of many human tissues (Finne et al., 1983). Consequently, the CPs of this bacterium are not suitable antigens, and alternative protein vaccine candidates should be identified for the development of *E. coli* K1 vaccines.

Outer membrane protein A (OmpA) is an abundant protein that localizes to the bacterial outer membrane of *E. coli* K1 (Krishnan and Prasadarao, 2012). In addition to its biophysical role as a receptor for bacteriophages and bacteriocins, increasing evidence has shown that OmpA of *E. coli* K1 contributes greatly to its pathogenesis. OmpA has been demonstrated to be responsible for bacterial survival in blood via the evasion of complement attack and suppression of immune cells (Confer and Ayalew, 2013). More importantly, OmpA mediates adhesion to and penetration of HBMECs, a key step in the induction of meningitis (Xie et al., 2004). Bioinformatics analysis has shown that OmpA generally forms two domains: OmpA_TM_ (transmembrane domain) and OmpA_per_ (periplasmic domain) (Krishnan and Prasadarao, 2012). Most functions of OmpA depend on OmpA_TM_, which forms an 8-stranded antiparallel β-barrel structure with four long flexible loops (Pautsch and Schulz, 2000; Cierpicki et al., 2006). OmpA_per_ localizes to the periplasmic space and maintains the integrity of the cell wall by binding to peptidoglycan (Wang et al., 2016).

Theoretically, OmpA is expected to be a good target due to its localization, abundance, and contribution to pathogenesis, as noted above. Notably, OmpA-specific antibodies and synthetic peptides representing extracellular loop1 and loop2 of the protein significantly prevent the invasion of *E. coli* K1 into HBMECs (Prasadarao et al., 1996). In addition, *E. coli* K1 pre-incubated with recombinant OmpA shows reduced astrocyte activation and neutrophil infiltration (Wu et al., 2009). A recent study also showed that an OmpA inhibitor peptide was able to prevent the adhesion of *Acinetobacter baumannii, Pseudomonas aeruginosa*, and *E. coli*, and thereby conferred protection in a murine sepsis peritoneal model (Vila-Farrés et al., 2017). OmpA homologs from other Gram-negative bacteria have already been tested as vaccines. For instance, phase II clinical trials of a *P. aeruginosa* vaccine, IC43, containing a portion of OprF (an OmpA homolog), have recently been completed (Rello et al., 2017). Additionally, OmpA homologs from *E. coli* O157:H7 (Novinrooz et al., 2017), *Acinetobacter baumannii* (Zhang et al., 2016), and *Brucella abortus* (Simborio et al., 2016) have been reported to confer protections in animals.

However, to the best of our knowledge, few studies have addressed the potential of OmpA-based vaccines for *E. coli* K1 control, possibly due to the difficulty of producing the hydrophobic full-length OmpA of OmpA_TM_ in water. To address this issue, we first examined the immunoreactivity of the four extracellular loops of OmpA. Then, we rationally designed and produced a soluble recombinant protein (OmpAVac) based on the structure of OmpA. The immune response and protective efficacy resulting from OmpAVac immunization were also investigated.

## Materials and methods

### Mice and strains

Three- to four-week-old specific pathogen-free female C57BL/6 mice were purchased from the Beijing HFK Bioscience Limited Company (Beijing, China). The mice were maintained under barrier conditions in a biohazard animal room. Approval for animal work was obtained from the Laboratory Animal Welfare and Ethics Committee CALAS of China (CALAS ID:20160376CQ03). The study protocol on human samples was reviewed and approved by the Animal Ethical and Experimental Committee of the Third Military Medical University in China (No. TMMU001018). The written informed consent was obtained from each donor of serum samples. Strains and plasmids used in this study were summarized in Table S1.

### Evaluation of the immunogenicity of OmpA loops

*E. coli* K1-infected patients and healthy donors were recruited from Southwest Hospital in Chongqing, China. Peptides corresponding to loop1, loop2, loop3, and loop4 were synthesized and conjugated to keyhole limpet hemocyanin (KLH) by the Chinapeptides Corporation (Shanghai, China). An ELISA was applied to test the levels of anti-loop antibodies in sera from recovered patients. Briefly, 96-well plates were coated with 0.8 μg of the peptides, which were then blocked with 2 mg/mL of BSA. Sera (diluted 1:500) from the eight donors were then added, and the plates were incubated at 37°C for 60 min. Then, the plates were washed three times, and horseradish peroxidase (HRP)-labeled goat anti-human Fc antibodies (Abcam) were added, followed by incubation at 37°C for 40 minutes. Color was developed with the 3, 3, 5, 5-tetramethylbenzidine (TMB, Sigma) substrate, and the optical density at 450 nm was measured by Varioskan™ LUX multimode microplate reader following manufacture's instruction.

As recombinant OmpA_TM_ is insoluble in water, OmpA_TM_ fused with maltose binding protein (MBP-OmpA_TM_) was produced from *E. coli* BL21. Briefly, the gene encoding OmpA_TM_ was synthesized and cloned into the pMal-c5x vector (Riggs, 2001). Then, the recombinant plasmid was transformed into *E. coli* BL21, and protein expression was induced with 0.5 mM IPTG for 4 h at 37°C. The cells were then collected and homogenized via sonication for 20 min. After centrifugation, the supernatant was collected and incubated with amylose resin on ice for 3 h. MBP-OmpA_TM_ was eluted from the resin with 20 mM maltose in PBS. Then MBP-OmpA_TM_ was concentrated and loaded onto G25 desalting column in PBS buffer for the removal of maltose.

MBP-OmpA_TM_ and the OmpA loops conjugated with KLH were purified and used to immunize 6- to 8-week-old mice. Five mice in each group were immunized three times at 7-day intervals. Each mice received an intramuscular injection 50 μg of protein formulated with 50 μg of Al(OH)3 in 100 μl of PBS buffer. Immunization of PBS was used as negative control. Ten days after the last immunization, sera were collected and stored at −80°C until further use. The titer of loop-specific antibodies was determined via ELISA as previously described (Gao et al., 2017). As an additional step, the sera were pre-incubated with the MBP protein or KLH at 37°C for 60 min to remove any non-specific reaction products.

### Production of OmpAVac constructs

**Figure 1:**
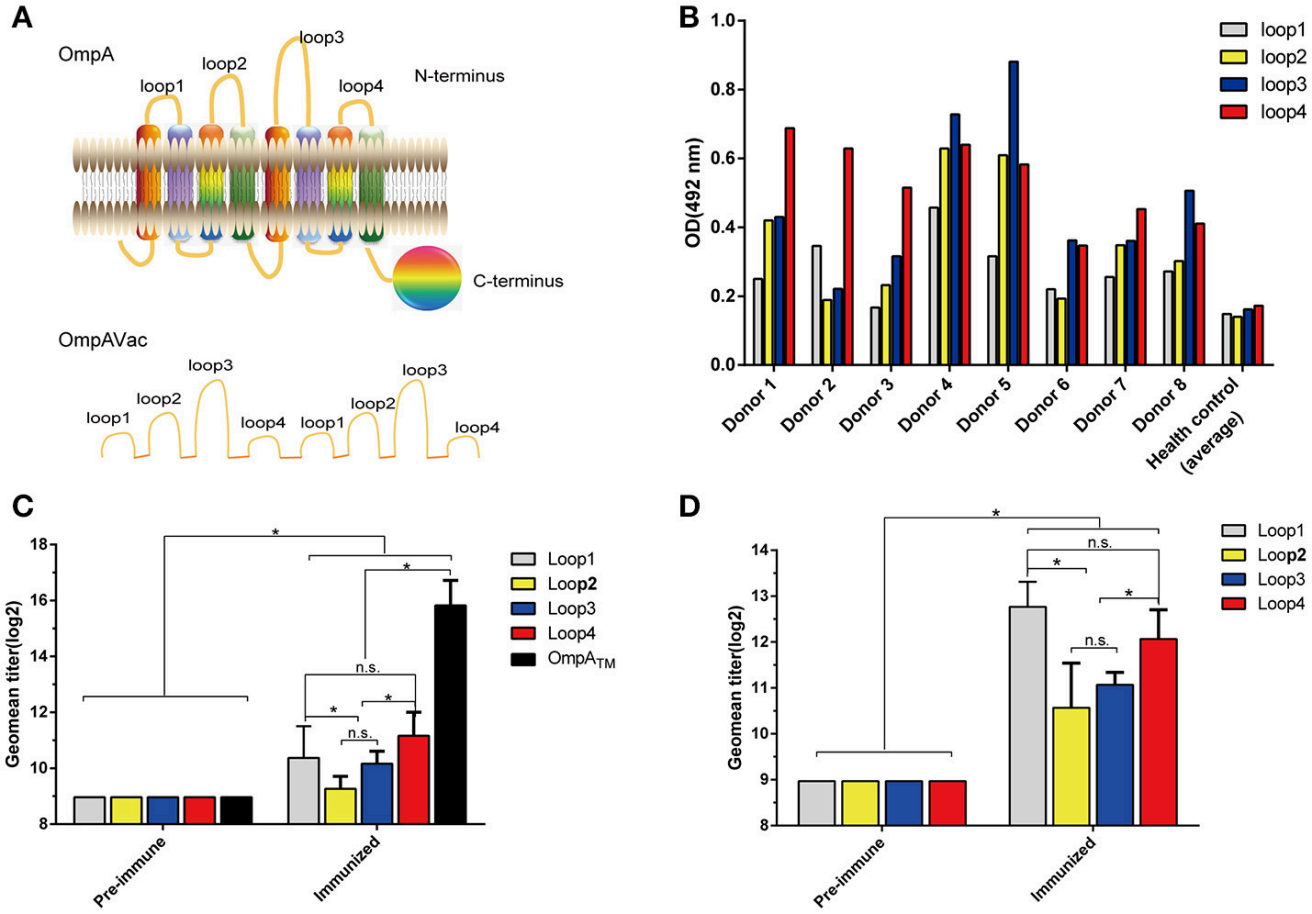
Rational design of OmpAVac. **(A)** Schematic representation of OmpA (upper) and OmpAVac (lower). **(B)** Reactions of loop1, loop2, loop3 and loop4 of OmpA with sera from *E. coli* K1-infected patients. The optical density (OD) from ELISAs of each patient donor and the average of 10 health donors was shown. **(C)** Evaluation of the immunogenicity of loop1, loop2, loop3, and loop4 in the form of OmpA_TM_ (transmembrane domain of OmpA) fused with MBP (maltose binding protein) tag. The titers of anti-loop1, anti-loop2, anti-loop3, and anti-loop4 antibodies from MBP-OmpA_TM_-immunized mice are shown. **(D)** Evaluation of the immunogenicity of loop1, loop2, loop3, and loop4 in the form of synthesized peptides. Mice were immunized with synthesized peptides encoding loop1, loop2, loop3, or loop4 of OmpA. The titers of the anti-loop1, anti-loop2, anti-loop3, and anti-loop4 antibodies are shown. The significance of the differences was determined by unpaired parametric tests (Student's *t*-test for two groups or one-way ANOVA for three or more groups). ^*^indicates a significant difference when *P-*value is below 0.05, while “n.s.” indicates no significant difference.

**Figure 2:**
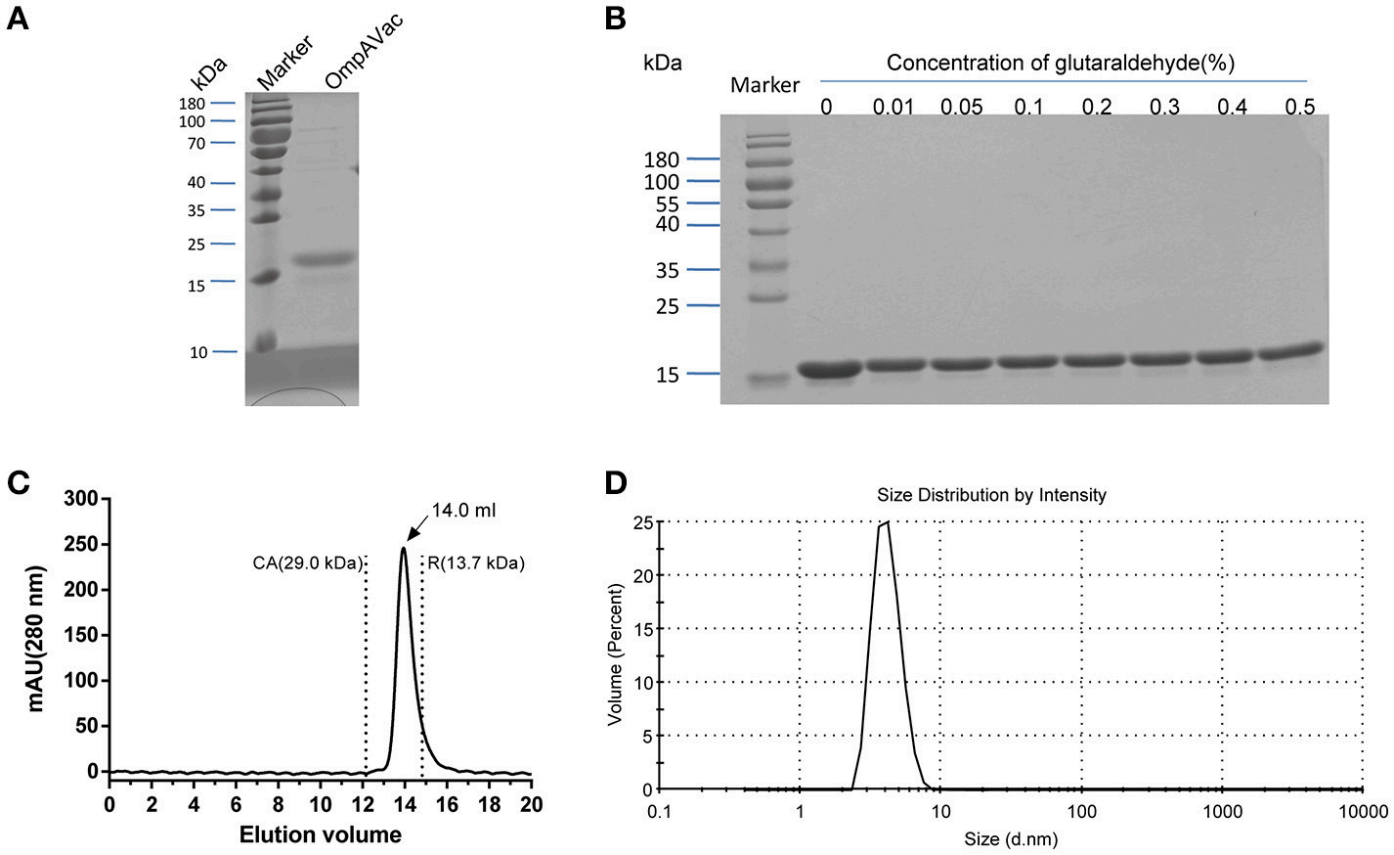
Characterization of purified OmpAVac. **(A)** SDS-PAGE analysis of OmpAVac. The purity of OmpAVac was ~93.2%, as determined based on the density of the corresponding band in an SDS-PAGE gel. **(B)** Cross-linking assay of OmpAVac. The concentrations of glutaraldehyde in lanes 1–8 were 0, 0.01, 0.05, 0.1, 0.2, 0.3, 0.4, and 0.5%, respectively. No oligomers or aggregates were observed. **(C)** Chromatography analysis of OmpAVac. OmpAVac produces a symmetrical peak at 14.0 mL, and the elution volumes of the protein standards CA (carbonic anhydrase) and R (ribonuclease A) were 12.2 and 13.7 mL, respectively. **(D)** Dynamic light-scattering analysis of OmpAVac resulted in a symmetrical peak with a diameter of 3.8 nm.

OmpAVac comprised two repeats of loop1-loop2-loop3-loop4 linked by a flexible Gly-Ser-Gly-Gly-Ser-Gly linker (Figure 1A and Figure S1). The nucleotide sequence encoding OmpAVac was synthesized and cloned into pGEX-6p-1 (GE Healthcare) using the BamH I and Xho I restriction sites. The expression of GST-tagged OmpAVac in *E. coli* BL21 was induced by adding 0.3 mM IPTG for 10 h in LB medium when the OD600 reached ~0.6. The cells were collected and lysed in lysis buffer (20 mM Tris, pH 8.0, 250 mM NaCl) via sonication. Then, the cell lysate was centrifuged at 12,000 rpm for 30 minutes, and the supernatant was collected and incubated with glutathione resin (NEB). After extensive washing, Precision protease (GE Healthcare) was added, followed by incubation at 4°C for 10 h to remove the GST tag. OmpAVac was then eluted and loaded onto a Resource Q column (GE Healthcare) in buffer (20 mM Tris, pH 8.0, 50 mM NaCl). OmpAVac was then eluted with a gradient (10 column volume in 30 minutes) of high salt buffer (20 mM Tris, pH 8.0, 500 mM NaCl). Finally, the purified OmpAVac was concentrated to ~4–5 mg/mL in buffer (PBS, pH 7.2) by Amicon™ Ultra centrifugal filters (Millipore) and stored at−80°C for future use. OmpAVac was confirmed via protein N-terminal sequencing and quantified via SDS-PAGE analysis (Figure 2A).

### Determination of the oligomerization and homogeneity of OmpAVac

First, size-exclusion chromatography was applied to determine the oligomerization of purified OmpAVac as previously described (Gao et al., 2017a), with slight modifications. The standard proteins Blue dextran 2000, aldolase, conalbumin, ovalbumin, carbonic anhydrase (CA), and ribonuclease A (R) were purchased from GE Healthcare. OmpAVac and the protein standards were diluted to 10 mg/mL and loaded onto a Superdex 75/300 column in buffer (20 mM Tris, pH 8.0). The elution peaks of OmpAVac and the protein standards were recorded. The predicted Mw of OmpAVac was calculated as described by Haiguang et al. (Wang et al., 2016). For the dynamic light-scattering assay, purified OmpAVac was diluted to 0.5 mg/mL and loaded onto a Zetasizer (Malvern, UK) equipped with an argon ion laser. The analysis was then performed three times at 25°C.

The cross-linking reaction of OmpAVac was performed using glutaraldehyde as a linking agent (Fadouloglou et al., 2008). In brief, 30 ng/mL OmpAVac was incubated with glutaraldehyde at 4°C for 10 h. The final concentration of glutaraldehyde in each reaction was adjusted to 0.05, 0.1, 0.2, 0.3, 0.4, and 0.5. The reaction was stopped by the addition of SDS loading buffer. Finally, the protein samples were visualized via SDS-PAGE.

### Mouse immunization and challenge

In each group, 25 mice were subcutaneously immunized with 100 μl of vaccine on days 0, 7, and 14. Each 100 μl of vaccine contains 25 μg of OmpAVac or KLH-conjugated peptide (loop1, loop2, loop3, and loop4) formulated with 50 μg of aluminum adjuvant. Immunizations with KLH and PBS alone were performed as negative controls. Five mice from each group were sacrificed on day 21, and their sera were collected to determine the titer of antigen-specific antibodies. In addition, splenic cells were isolated to evaluate cellular immunity.

To investigate the protection elicited by OmpAVac vaccination, 10 mice in each group were intraperitoneally injected with a lethal dose (1 × 10^8^ CFU per mice) of *E. coli* K1 RS218 in 250 μL of sterilized PBS. The number of deaths was recorded daily for 14 days. For animal welfare, Pre-emptive euthanasia was given to mouse in moribund states, such as paralysis, hyperspasmia, no response to external stimuli and other signs. These mice were given euthanasia and recorded as death. To further investigate the protective mechanism, another five mice were intraperitoneally challenged with a sublethal dose (1 × 10^7^ CFU per mice) of *E. coli* K1. Their body weight was monitored daily for 14 days, and the percentage of the initial weight was calculated. In addition, five immunized mice in each group were sacrificed at 24 h after challenge as previously described, and the bacterial loads in their blood and spleen were determined. The spleens of the sacrificed mice were collected, weighed, and homogenized in 1 mL of sterilized PBS buffer. The spleen homogenates and blood were plated onto LB plates at a 10-fold serial dilution and cultured at 37°C for 20 h. The number of colonies on the plates was counted and used to calculate the bacterial load. The CFU per gram of tissue was calculated for the comparison of bacteria load.

### ELISA

ELISA was used to evaluate the humoral immune response elicited by OmpAVac vaccination. In brief, flat-bottomed 96-well ELISA plates were coated with purified OmpA (200 ng/well) or synthesized peptides encoding loop1, loop2, loop3, and loop4 (600 ng/well) and incubated overnight at 4°C. Then, the antigen-coated plates were blocked with 200 μL of blocking buffer [PBS containing 0.05% Tween 20 and 2% BSA, pH 7.5 (PBST)] for 60 minutes at 37°C. Serially diluted (two-fold) sera (100 μL) were then added to each well, followed by incubation for 60 min at 37°C. After rinsing three times with PBST, the plates were incubated with HRP-conjugated anti-mouse Fc antibodies (Abcam) for 1 h at 37°C. Finally, color was developed with TMB (Sigma), and the reaction was stopped by adding 0.1 M sulfuric acid. The optical density (OD) was determined at 450 nm. The subtype of anti-OmpAVac IgG was also determined via ELISA. The major difference in these assays was that the sera from OmpAVac-immunized mice were diluted 1:2000 and used as primary antibodies, while goat anti-mouse IgG1, IgG2a, and IgG2b mAbs (Abcam) were used as secondary antibodies. The remaining steps were the same as those described for determination of the titer of OmpAVac antibodies.

### Splenic cell proliferation and cytokine assays

The stimulation index (SI) was measured to detect the splenic immune response as described before (Yang et al., 2017). Total spleen cells were collected and separated into single cells by cell strainer. Subsequently, the cells were aliquoted into 96-well plates (1 × 10^6^ cells/well) and cultured in DMEM containing 10% FBS (HyClone). Then, 5 μg of OmpAVac was added, followed by co-incubation for 36 h to stimulate the spleen cells. Cells treated with 10 mM phytohemagglutinin-A (PHA) (Gibco) and unstimulated cells served as positive and negative controls, respectively. Cell proliferation was determined using cellular incorporation of 5-bromo-2-deoxyuridine (BrdU, Roche, Germany), followed by absorbance measurements (Ab) at 450 nm. The SI was calculated by dividing the Ab of stimulated cells by the Ab of unstimulated cells. Additionally, the supernatant was collected to measure the concentrations of IL-4, INF-gamma, and IL-17A via ELISA according to the manufacturer's instructions (R&D Systems).

### *In vivo* evaluation of the protection conferred by OmpAVac-specific antibodies

To prepare OmpAVac-specific antibodies, purified OmpAVac formulated with aluminum adjuvant was used to immunize rabbits three times, on days 0, 21, and 28. Sera were collected from the rabbits, and IgG was purified with a Protein G column (GE Healthcare) by affinity chromatography according to the manufacturer's instructions. The purified antibodies were quantified via the BCA assay and non-reducing SDS-PAGE analysis. Sera from unimmunized rabbits were also collected, and IgG was purified as previously described. Three- to four-week-old C57BL/6 mice were intraperitoneally administered purified OmpAVac-specific antibodies at 12 h prior to challenge. The doses of the antibodies in the high, medium, and low groups were 3, 1, and 0.3 mg per mouse, respectively. As a control, mice were administered with 3 mg of IgG from an unimmunized rabbit per mouse. The remaining steps for the infection of mice were the same as those described in the previous paragraph. In addition, the protective effects of OmpA-specific antibodies were evaluated in neonatal mice. In brief, ten 3-days old mice in each group were administered intraperitoneally with three different dose of antibodies (3, 1, and 0.3 mg per mouse, respectively). Twenty four hours later, mice received an intraperitoneal inoculation of 10^2^ CFU of *E. coli* K1 RS218. The number of death was monitored daily for 7 days.

### Opsonophagocytic killing assay

The opsonophagocytic killing assays were performed as described by Yang et al. (2017). Briefly, peripheral promyelocyte HL-60 cells (ATCC CCL-240) were differentiated into granulocyte-like cells with the addition of 100 mM N′,N dimethylformamide (Sagon) to the growth medium for 4 days. Then, 4 × 10^5^ HL-60 cells (in a volume of 40 μL), 10^3^ CFU of *E. coli* K1, and 20 μL of diluted serum or PBS (control) were added to 96-well plates. In each well, 10 μL of 1% infant rabbit serum was added as a source of complement (Pel-Freez). These mixtures were co-incubated at 37°C for 3 h and then plated onto agar medium. The number of bacterial colonies on the plates was counted, and bactericidal activity in the sera was calculated using the following formula: (N_PBS_-N_sera_)/N_PBS_ × 100%, where N_PBS_ and N_sera_ refer to the number of bacteria survived in HL-60 cells treated with PBS and sera, respectively. Serum from recombinant MBP-OmpA_TM_-immunized mice was used as a positive control.

### Attachment and invasion inhibition assay

The attachment and invasion assays were performed as described by Prasadarao et al., with slight modifications (Prasadarao et al., 1996). Briefly, BMECs (CD31^+^)were cultured on collagen-coated 24-well tissue culture plates and incubated at 37°C for ~5 days. *E. coli* K1 RS218 cells in the log phase of growth were incubated with antibodies or medium on ice for 1 hour. Then, ~10^7^ bacteria were added to the BMEC monolayers at a ratio of infection (ROI) of ~1:100. Subsequently, the culture plates were incubated for 2 h at 37°C in 5% CO_2_ without shaking. After washing four times with pre-warmed M199 medium, the cell-associated bacteria were released using lysis buffer with 0.5% Triton X-100 and plated onto LB agar. For the quantification of invaded bacteria, the monolayers were treated with 100 μg/ml of gentamicin at 37°C for 1 hour to kill attached bacteria. For the quantification of total associated bacteria, the antibiotics-free medium instead of gentamicin was used to treat monolayers. Then the released bacteria were enumerated by plating onto LB agar. Each assay was performed in triplicate and repeated at least three times.

### Statistical analysis

The data are presented as the means ± SE. The significance of the differences was determined by unpaired parametric tests (Student's *t*-test for two groups or one-way ANOVA for three or more groups). The significance of the differences of bacteria load was determined by unpaired nonparametric tests (Mann Whitney test). The Kaplan-Meier test was employed for analysis of the survival rate. SPSS13.0 (SPSS Software) was used for data analyses. Differences were considered significant when the *P-*value was <0.05. All experiments, except for animal challenge assays, were conducted a minimum of three times.

## Results

### The loops of OmpA are immunogenic and immunoreactive in multiple forms

To test the immunogenicity of the extracellular loops of OmpA, firstly sera from eight *E. coli* K1-infected patients were collected, and their contents of anti-loop antibodies were determined via ELISA. Notably, antibodies against loop1, loop2, loop3, and loop4 were not observed in sera from the healthy control but were observed in sera from all *E. coli* K1-infected patients (Figure 1B). However, no significant difference in the response was noted among loop1, loop2, loop3, and loop4. Next, to verify whether these loops are immunogenic in the form of a recombinant protein, recombinant MBP-OmpA_TM_ was purified and used to immunize mice. As shown in Figure 1C, the titer of antibodies against OmpA_TM_ and the four separated loops increased sharply compared with that in pre-immune sera. In addition, the titers of anti-loop1 and anti-loop4 antibodies were significantly higher than those of anti-loop2 and anti-loop3 antibodies, indicating the stronger immunogenicity of loop1 and loop4. However, no significant difference of titer was observed between anti- loop1 and anti- loop4. Similarly, loop-specific antibodies were obviously detected after immunization with the synthesized loops (Figure 1D), suggesting that the separate loops are immunogenic. Taken together, these findings indicate that all four extracellular loops of OmpA are immunogenic and immunoreactive in their native state, recombinant protein and separated loop forms.

### Rational design, production, and characterization of OmpAVac

Building on the evidence showing that the four extracellular loops were immunogenic, we next combined the loops to generate a vaccine with improved efficacy. After several attempts, the four loops were successfully connected together, and OmpAVac was generated (Figure 1A, lower panel), of which the sequence was shown in figure S1. OmpAVac comprised two repeats of loop1-linker-loop2-linker-loop3- -linker-loop4, with a the flexible linker sequence of Gly-Ser-Gly-Gly-Ser-Gly. Single copy of loops 1-4 was also be tested, however it precipitated on the resin after removal of GST tag, which hindering its further evaluation. Fortunately, ecombinant OmpAVac remains soluble after cleavage of the GST tag (Figure 2A). As homogeneity of a protein is critical for its further application in vaccine development, we next determined the oligomerization of OmpAVac using three methods. First, the results of chemical cross-linking indicated that OmpAVac performs as a monomer, as no dimers or higher oligomer forms of the protein were observed with an increase in glutaraldehyde (Figure 2B). Moreover, the elution volume of OmpAVac from the Superdex 75 column was ~14.0 mL. The molecular weight was predicted to be ~16.2 kDa, according to the elution volumes of the standard protein markers. Consistent with the results of size-exclusion chromatography, the dynamic light-scattering analysis showed that the diameter of OmpAVac was 3.8 nm, and its calculated molecular weight in solution was ~15.6 kDa (Figure 2C). Overall, the results indicate that recombinant OmpAVac behaves as a homogenous monomer in solution.

### OmpAVac induces a multifactorial immune response in mice

**Figure 3:**
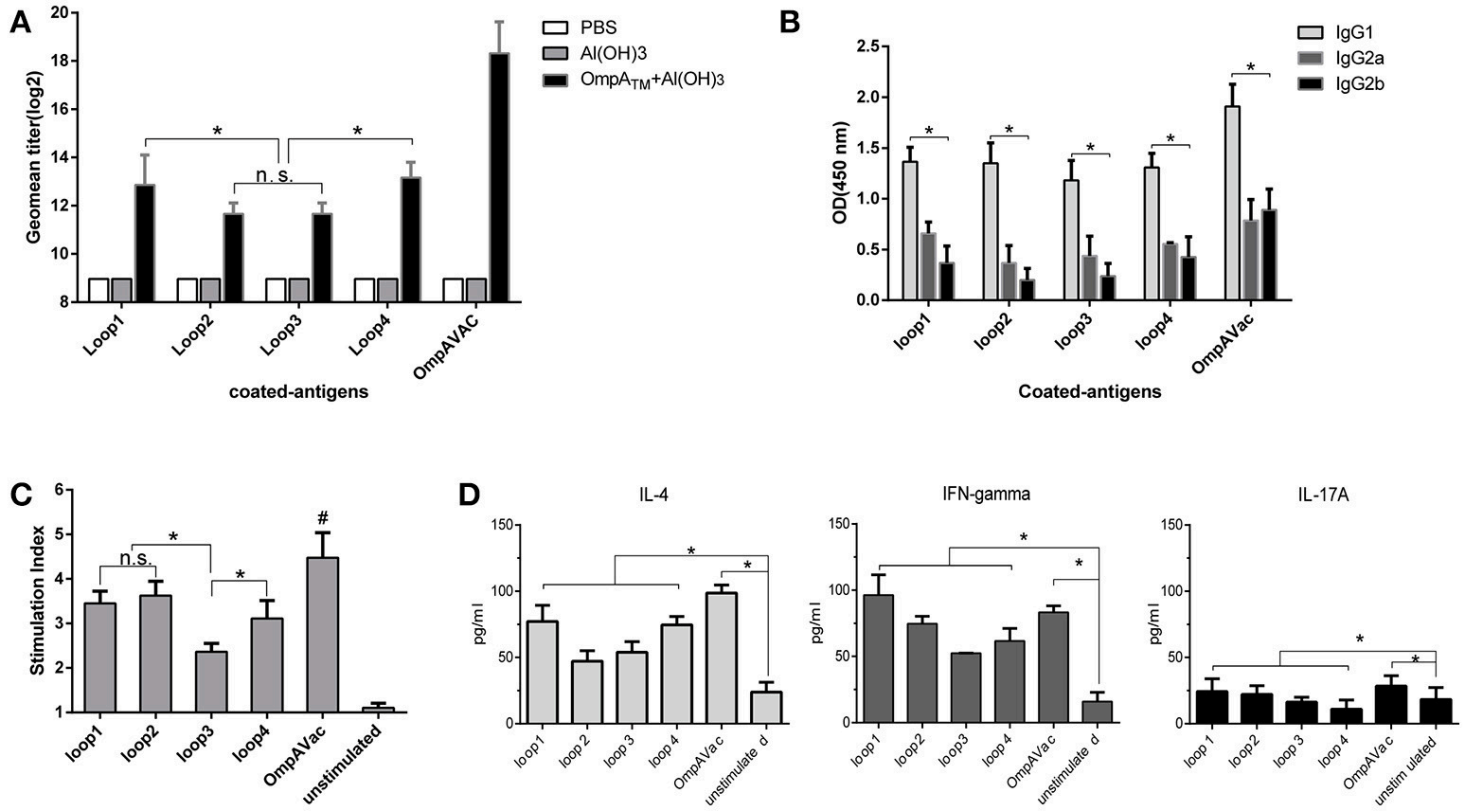
OmpAVac induces a multifactorial immune response in mice. **(A)** The bar represents the titer of anti-loop1, anti-loop2, anti-loop3, anti-loop4, and anti-OmpAVac IgGs from OmpAVac-immunized mice. ^*^indicates a significant difference (*P* < 0.05), while “n.s.” indicates no significant difference. **(B)** Comparison of the subtypes of anti-loop1, anti-loop2, anti-loop3, anti-loop4, and anti-OmpAVac IgGs from OmpAVac-immunized mice. The OD at 450 nm in each ELISA reaction is shown. ^*^indicates a significant difference in the OD among IgG1, IgG2a, and IgG2b. **(C)** Proliferative activity of mouse splenocytes after *in vitro* stimulation with loop1, loop2, loop3, loop4, and OmpAVac for 72 h, respectively. Proliferation was measured using the bromodeoxyuridine (BrdU) labeling method. ^*^indicates a significant difference, while “ns” indicates no significant difference. “#” indicates a significant difference between the OmpVac-stimulated group and the other groups. **(D)** Cytokine production by antigen-stimulated splenocytes from OmpAVac-immunized mice. Two weeks after the final immunization, the spleens were processed and stimulated with loop1, loop2, loop3, loop4, and OmpAVac for 72 h, and the levels of IL-4, IFN-gamma, and IL-17 in each culture supernatant were measured. The data are shown as the means ± SE and the significance of the differences was determined by unpaired parametric tests (Student's *t*-test for two groups or one-way ANOVA for three or more groups). ^*^indicates a significant difference (*P* < 0.05).

To further verify whether OmpAVac could serve as a vaccine candidate for *E. coli* K1, we characterized the immune response of mice after OmpAVac vaccination. As expected, a sharp increase in the level of OmpAVac-specific IgG antibodies was detected in sera from immunized mice compared with that in sera from pre-immune mice (Figure 3A). More importantly, antibodies against loop1, loop2, loop3, and loop4 were detected in OmpAVac-immunized mice, indicating that the immunogenicity of all four loops was preserved in the recombinant OmpAVac. Similar to the results of vaccination with the separate loops (Figure 1D), the titers of anti-loop1 and anti-loop4 IgG were higher than those of anti-loop2 and anti-loop3 (*P* < 0.05), providing additional evidence of the stronger immunogenicity of loop1 and loop4 compared with loop2 and loop3 (Figure 3A). In addition, the major subtype of OmpAVac-elicited antibodies was not IgG2a or IgG2b but rather IgG1 (Figure 3B), indicating a Th2-predominant response against OmpAVac vaccination.

We next verified cellular immunity against OmpAVac via spleen proliferation experiments. As shown in Figure 3C, the introduction of OmpAVac significantly increased the SI (stimulation index) of spleen cells from OmpAVac-immunized mice. Incubation of the spleen cells with the separate loops had a similar effect. The SIs were higher for loop1, loop2, and loop4 than for loop3, indicating that more predominant T-cell epitopes are present in these three loops. Additionally, the concentrations of IL-4, IFN-γ, and IL-17A in the supernatants were measured to verify the type of immune response. Consistent with the results of IgG subtyping, an obvious Th2 response was observed, as the level of IL-4 increased dramatically after stimulation with OmpAVac or the OmpA loops (Figure 3D). The secretion of IL-17A was also increased, suggesting a modest Th17 reaction elicited by OmpAVac vaccination.

### OmpAVac vaccination confers protection against *E. coli K1* infection

**Figure 4:**
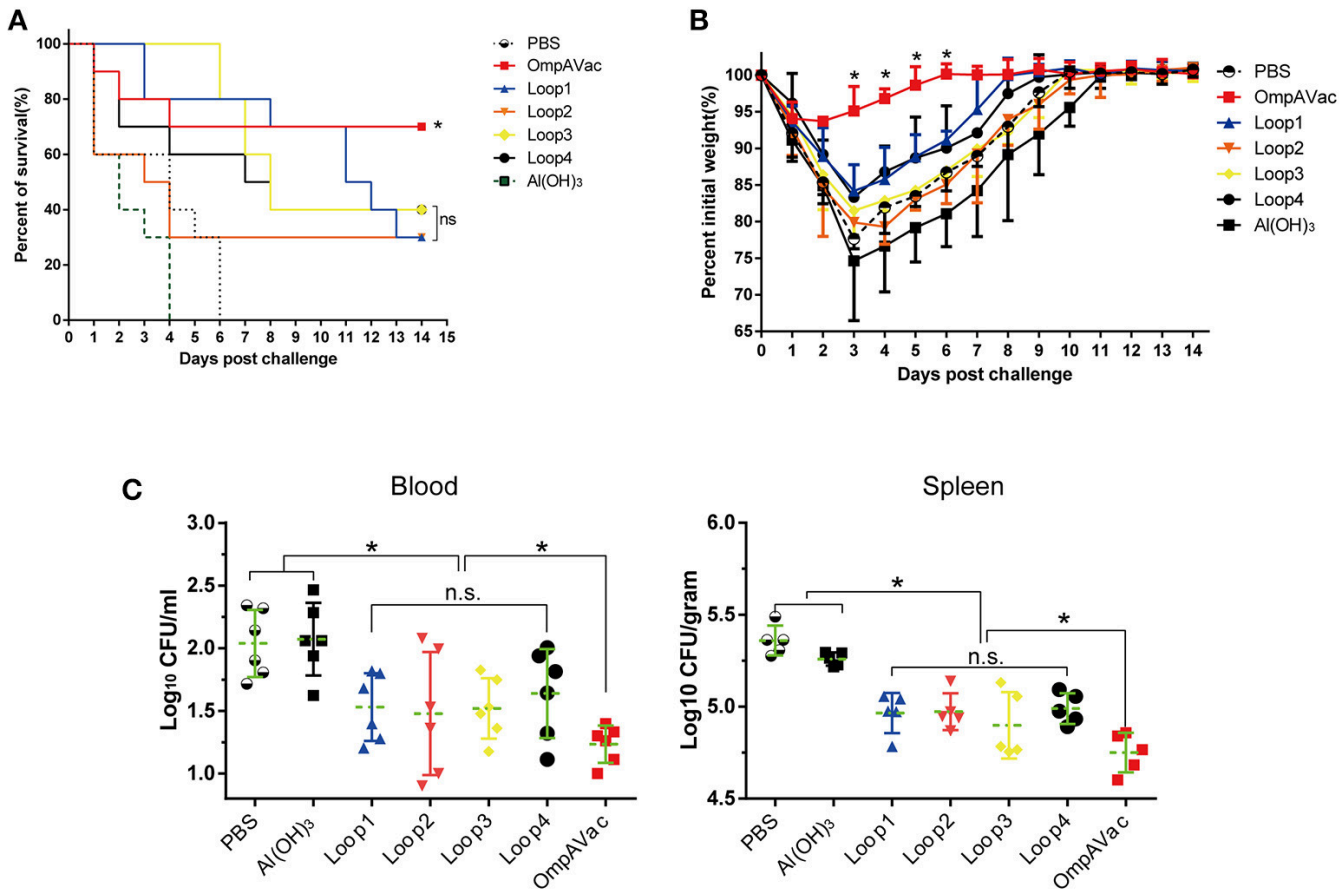
OmpAVac vaccination confers protection against *E. coli K1* infection. **(A)** Survival rates of mice challenged with a lethal dose of *E. coli* K1 RS218. Ten mice in each group were immunized with loop1, loop2, loop3, loop4, and OmpAVac 10 days prior to challenge. The number of survivors was recorded daily for 14 days. The Kaplan-Meier test was employed for analysis of the survival rate. ^*^indicates a significant difference between the OmpVac-immunized group and the other groups. “ns” indicates no difference among loop1-, loop2-, loop3-, and loop4-immunized groups. **(B)** Change in the weights of immunized mice challenged with a sublethal dose of *E. col*i K1 RS218. The weight of each mouse was recorded daily for 14 days. The percentage of their initial weight is shown. ^*^indicates a significant difference between the OmpAVac group and the remaining groups. **(C)** The bacterial load in the blood and spleen of immunized mice at 24 h after challenge with a sublethal dose of *E. coli* K1 RS218. The log values of the number of bacteria per mL of blood or gram of spleen are shown. The significance of the differences of bacteria load was determined by unpaired nonparametric tests (Mann Whitney test). ^*^indicates a significant difference (*P* < 0.05), while “n.s.” indicates no significant difference. The data are presented as median and interquartile ranges.

In this setting, immunized mice were challenged with *E. coli* K1 to assess the protection conferred by OmpAVac vaccination. The mice immunized with OmpAVac exhibited significantly better survival than any of the other groups (Figure 4A), showing a mortality rate of 30%. Moreover, restricted protection was observed in loop1-, loop2-, loop3-, and loop4-immunized mice, but no significant differences were observed among the groups (Figure 4A). A similar trend of protection was observed in the sublethal *E. coli* K1 infection model. First, there were clear differences in weight loss and recovery among the groups after sublethal *E. coli* K1 challenge (Figure 4B). OmpAVac-immunized mice showed the smallest weight reduction, which returned to a healthy level in no more than 6 days. However, the loop-immunized mice showed greater losses of body weight and recovered slowly. Second, the number of bacteria in the blood and spleen was counted to further clarify the protection conferred by OmpAVAC immunization. The results showed a marked reduction of the bacterial load in the lungs and blood from OmpAVac-immunized mice (Figure 4C). In addition, a decrease in bacterial colonization was observed in loop1-, loop2-, loop3-, and loop4-immunized mice compared with the PBS and adjuvant controls. These data collectively indicate that OmpAVac vaccination confers effective protection against *E. coli K1* infection.

### Anti-OmpAVac antibodies contribute to OmpAVac-mediated protection

**Figure 5:**
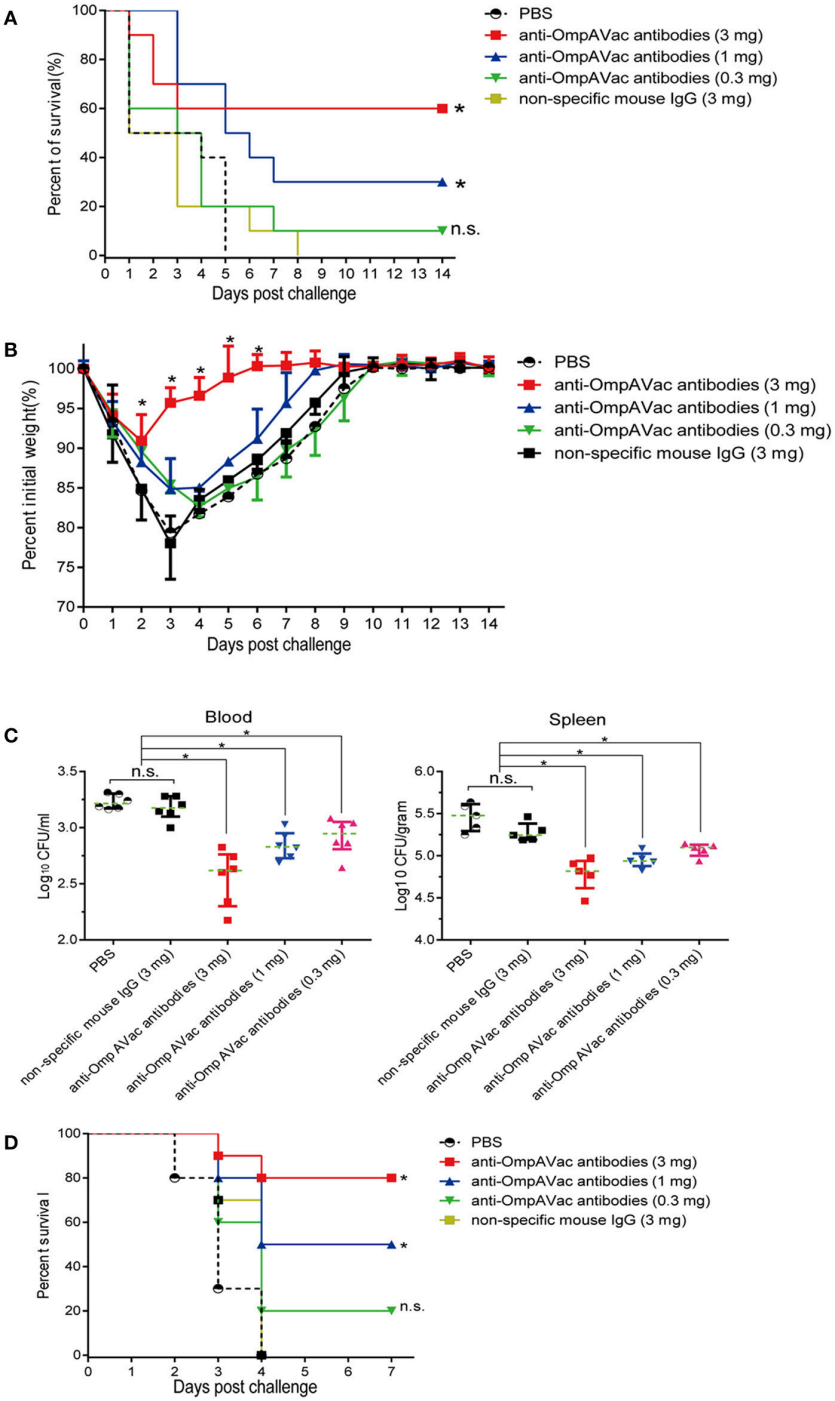
Anti-OmpAVac antibodies contribute to OmpAVac-mediated protection. **(A)** Survival rates of mice challenged with a lethal dose of *E. coli* K1 RS218. Ten mice each in each groups were administered 3, 1, and 0.3 mg of anti-OmpAVac antibodies, respectively. Twenty-four hours later, the mice were challenged with a lethal dose of *E. coli* K1 RS218. The number of survivors was recorded daily for 14 days. Three mg of IgG purified from unimmunized mice was used as a negative control. The Kaplan-Meier test was employed for analysis of the survival rate. ^*^indicates significant difference between vs PBS control group and non-specific mouse Ig group (*P* < 0.05), while “n.s.” indicates no significant difference (*P* > 0.05). **(B)** Weight change in immunized mice challenged with a sublethal dose of *E. coli* K1 RS218. Mice were administered anti-OmpA IgGs at 24 h prior to challenge. The percentage of their initial weight is shown. The significance of the differences was determined by unpaired parametric tests (one-way ANOVA). ^*^indicates significant difference among the four five groups (*P* < 0.05). **(C)** The bacterial load in the blood and spleen of mice challenged with a sublethal dose of *E. coli* K1 RS218. Mice were administered anti-OmpA IgGs at 24 h prior to challenge. The log values of the number of bacteria per mL of blood or gram of spleen are shown. The significance of the differences of bacteria load was determined by unpaired nonparametric tests (Mann Whitney test). ^*^indicates a significant difference (*P* < 0.05), while “n.s.” indicates no significant difference (*P* > 0.05). The data are presented as median and interquartile ranges. **(D)** Survival rates of newborn mice challenged with a lethal dose of *E. coli* K1 RS218. Three-days old mice were administrated with three different dose of anti-OmpAVac antibodies 24 h before challenge. PBS and non-specific mouse IgG were used as control. The number of death was recorded daily for 4 days. The Kaplan-Meier test was employed for analysis of the survival rate. ^*^indicates significant difference between vs PBS control group and non-specific mouse Ig group (*P* < 0.05), while “n.s.” indicates no significant difference (*P* > 0.05).

Since OmpAVac vaccination induced a Th2-predominant immune response, we hypothesized that anti-OmpAVac antibodies are protective against *E. coli* K1 infection. To test this hypothesis, anti-OmpAVac antibodies were purified and administered to the mice prior to challenge with a lethal dose of *E. coli* K1. As shown in Figure 5A, a dose-dependent change in the survival rate was observed in mice administered anti-OmpAVac antibodies. However, no protective effect was observed in the non-specific mouse IgG and PBS control groups. Mice given 0.3 mg anti-OmpAVac antibodies showed no significant difference with PBS control, suggesting sufficient amount of antibodies was required to guarantee protection. In another experimental setting, the mice injected with a high dose (3 mg/mouse) of anti-OmpAVac antibodies showed the smallest weight loss recovery over a period as short as 6 days, and the middle- (1 mg/mouse) and low-dose groups (0.3 mg/mouse) also exhibited restricted improvement of health compared with the PBS and mouse IgG controls (Figure 5B). In addition, the bacterial load in the blood and spleen decreased with higher titers in anti-OmpAVac antibodies (Figure 5C). Finally, the protection of OmpAVac specific antibodies was accessed on neonatal mice infection model. Consistent with the trend observed from adult mice, a dose-dependent protection of anti- OmpAVac antibodies was observed (Figure 5D). Together, these results suggest that OmpAVac-specific antibodies confer protection against *E. coli* K1 infection.

### Anti-OmpAVac antibodies mediate opsonophagocytosis and inhibit bacterial attachment and invasion

**Figure 6:**
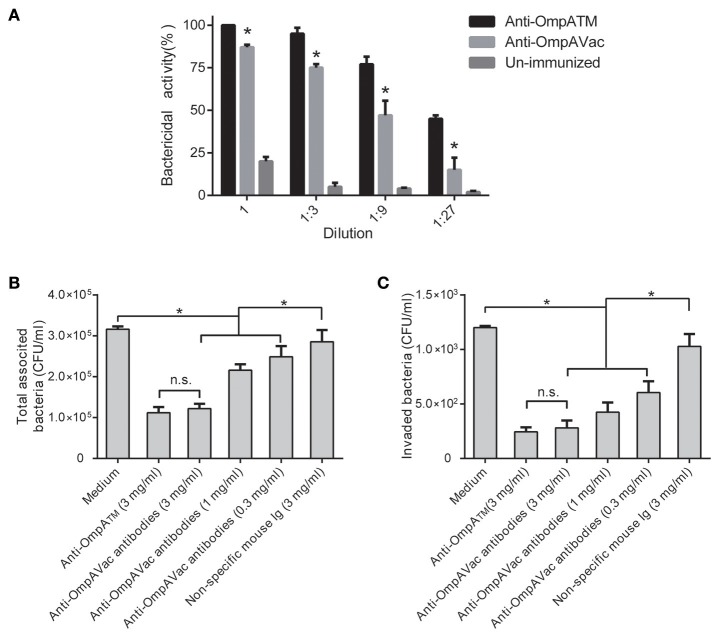
Anti-OmpAVac antibodies mediate opsonophagocytosis and inhibit bacterial attachment and invasion. **(A)** Opsonophagocytic assay of anti-OmpAVac antibodies. Sera from immunized mice were diluted and incubated with *E. coli* K1. The bar represents the percentage of killed bacteria in a series of dilutions. The data are presented as the means ± SE. Anti-OmpAVac antibodies showed marked bactericidal activity. ^*^indicates a significant difference between anti-OmpAVac group and the unimmunized group (*P* < 0.05). **(B)** Total associated bacteria treated with anti-OmpAVac antibodies. The bar represent the mean value plus the standard error of the number of total associated bacteria. **(C)** Bacterial invasion activity assays for anti-OmpAVac antibodies. The mean value plus the standard error of the number of bacteria invaded into human brain microvascular endothelial cells for each group is shown. Bacteria treated with medium was used as control. The unpaired Student's *t*-test was used to determine the significance of the differences between two groups. ^*^indicates a significant difference (*P* < 0.05) while “n.s.” indicates no significant difference.

To further elucidate the mechanism of anti-OmpAVac antibody-mediated protection, we first tested opsonophagocytic activities. As shown in Figure 6A, marked bactericidal activity of sera from both OmpAVac- and MBP-OmpA_TM_-immunized mice was observed, and MBP-OmpA_TM_-immunized sera showed significantly higher opsonophagocytic activity than sera from mice immunized with OmpAVac at the four tested dilutions. We next evaluated the effect of the OmpAVac-specific antibodies on the invasion of *E. coli* K1. The results showed that anti-OmpAVac antibodies significantly reduced attachment and invasion in a dose-dependent manner (Figures 6B,C), whereas mouse Ig had little effect on bacterial invasion. Taken together, these findings indicate that the observed anti-OmpAVac-mediated protection is likely due to opsonophagocytic activity and the inhibition of bacterial attachment and invasion.

## Discussion

Recent studies have shown that structural biology is playing an increasing role in vaccine design. Based on the 3D structure of proteins, it is now easier to generate antigens that are safe, immunogenic, broadly protective, stable, and druggable (Malito et al., 2015). For example, Maria et al. generated artificial factor H binding protein (fHBP) mutants according to the crystal structure of wild-type fHBP. These fHBP mutants induced broadly protective immunity because of an engineered surface with specificity for all fHBP variants (Scarselli et al., 2011). Another example is provided by the F protein of RSV, which is a good vaccine candidate but is difficult to produce due to its aggregation and low solubility in water. Through structural modeling, an engineered F protein was successfully generated via removal of the fusion peptide and the cytoplasmic domain. Herein, we rationally generated the recombinant OmpAVac protein after an in-depth analysis of the crystal structure of OmpA and confirmed that OmpAVac is a good candidate for the development of a vaccine against *E. coli* K1 infection. These data provide additional evidence that the structure-based design of vaccines using a combination of the extracellular loops of outer membrane proteins is possible.

One interesting finding of the present study was that the four extracellular loops of OmpA were immunogenic in the form of peptides as well as recombinant or native proteins. However, these loops cannot be used as vaccines due to their limited molecular weights. To rationally design an OmpA-based vaccine, we first combined the four loops using a flexible linker to maintain the structure of the separate loops. Second, we added a tandem repeat of the four loops to increase the size of the protein and obtain better immunogenicity. Thus, we successfully generated the soluble, homogenous and protective OmpAVac protein for the prevention of *E. coli* K1 infection. Notably, virus-like particles (VLPs), nanomaterials, inactive toxins, and other carriers can aid in the induction of more potent immunity by small peptides (Mrsny et al., 2002; Torres-Sangiao et al., 2016; Mohsen et al., 2017). Future studies may select an optimal carrier for OmpAVac to generate better protection.

Another interesting finding in our study was that anti-OmpA_TM_ antibodies increased the bactericidal activity, and decrease the invasion of BMEC compared to anti-OmpAVac antibodies. One explanation could be different structure of loops between MBP-OmpA_TM_ and OmpAVac. In form of OmpAVac, the loops was directly connect together without hydrophobic residues. Some conformation epitopes may not formed. Unlike OmpAVac, the whole transmembrane domain of OmpA was cloned, which allows the correct folding of conformational epitopes. As a result, OmpA_TM_ elicited more types of antibodies that recognize epitopes on native OmpA than OmpAVac. These data provided additional evidences that the critical contribution of the structure of an antigen to vaccine induced protection efficacy.

To fully evaluate the protective efficacy of OmpAVac, animal models of *E. coli* K1 induced-meningitis will be of high priority. *E. coli* K1 causes meningitis in neonatal mice and rats, and these models have been widely used to investigate the pathogenesis of *E. coli* K1 (Peng et al., 2012; Shanmuganathan et al., 2013; Witcomb et al., 2015). However, these *E. coli* K1 induced-meningitis models are not suitable for vaccine assessment because 2–3 weeks is required for active immunization. Thus, systematic infection models partially representing the pathogenic process of *E. coli* K1 infection (Dieelberg et al., 2012; McCarthy et al., 2015) were applied for the evaluation of OmpAVac vaccination. Future studies should focus on evaluating the protection conferred by OmpAVac-specific antibodies in animals with neonatal meningitis.

Young children are more susceptible to *E. coli* K1 infection because of their immature immune system. Thus, in addition to vaccines, therapeutic antibodies are an important tool for the control of *E. coli* K1 infection. *E. coli* K1 polysialic acid (PSA)-specific monoclonal antibodies have been reported to show bactericidal activities (Shin et al., 2001; Park et al., 2014). Anti-B polysaccharide antibodies transferred from mother rabbits to newborn rabbits also confer considerably more resistance to *E. coli* K1 infection (Lifely et al., 1989). Similarly, we observed that OmpAVac-specific antibodies mediated opsonization and inhibited bacterial invasion *in vitro*. More importantly, the transfer of anti-OmpAVac antibodies conferred significant protection in the examined both adult and neonatal infection models. These findings highlighted the potential utility of anti-OmpAVac antibodies in the treatment of *E. coli* K1 infection.

In summary, we showed that the four extracellular loops of OmpA are immunogenic, and we rationally generated the first recombinant OmpAVac according to the structure of OmpA. Vaccination with OmpA elicited multifactorial immune response and conferred protection in mice.

## Author contributions

HG, YL, JZ, and YW performed the major experiments. HG and YL wrote the draft. ZL and PC provided and analyzed the clinical samples. XW and QZ analyzed the data and revised the manuscript. JG designed the experiments and got the grants.

### Conflict of interest statement

The authors declare that the research was conducted in the absence of any commercial or financial relationships that could be construed as a potential conflict of interest.
