# Supplementary material for: Rational Design and Evaluation of an Artificial Escherichia coli K1 Protein Vaccine Candidate Based on the Structure of OmpA
Source: Front Cell Infect Microbiol. 2018 May 23;8:172. doi: 10.3389/fcimb.2018.00172 (PMC5974202; doi:10.3389/fcimb.2018.00172)
Supplement: Supplementary file 1 [file Table_1.DOCX]

Table S1 Plasmids and strains used in this study

| Name | Purpose | Reference |
| --- | --- | --- |
| Plasmids |  |  |
| pMal-c5x | Clone of OmpA_TM_ | ([Riggs, 2001](#_ENREF_2)) |
| pGEX-6P-1 | Clone of OmpAVac | ([Harper and Speicher, 2008](#_ENREF_1)) |
| pMal-c5x-OmpA_TM_ | Expression of MBP- OmpA_TM_ | This study |
| pGEX-OmpAVac | Expression of OmpAVac | This study |
|  |  |  |
| Strains |  |  |
| *E. coli* K1 RS218 | Infection of mice | ATCC 700973 |
| pMal-c5x/BL21 | Expression of MBP | This study |
| pMal-c5x-OmpA_TM_/BL21 | Expression of MBP- OmpA_TM_ | This study |
| pGEX-OmpAVac/BL21 | Expression of OmpAVac | This study |

Harper, S., and Speicher, D.W. (2008). Expression and purification of GST fusion proteins. *Curr Protoc Protein Sci* Chapter 6**,** Unit 6 6. doi:10.1002/0471140864.ps0606s52

Riggs, P. (2001). Expression and purification of maltose-binding protein fusions. *Curr Protoc Mol Biol* Chapter 16**,** Unit16 16. doi:10.1002/0471142727.mb1606s28
