# Supplementary figures and images for: Rational Design and Evaluation of an Artificial Escherichia coli K1 Protein Vaccine Candidate Based on the Structure of OmpA
Source: Front Cell Infect Microbiol. 2018 May 23;8:172. doi: 10.3389/fcimb.2018.00172 (PMC5974202; doi:10.3389/fcimb.2018.00172)

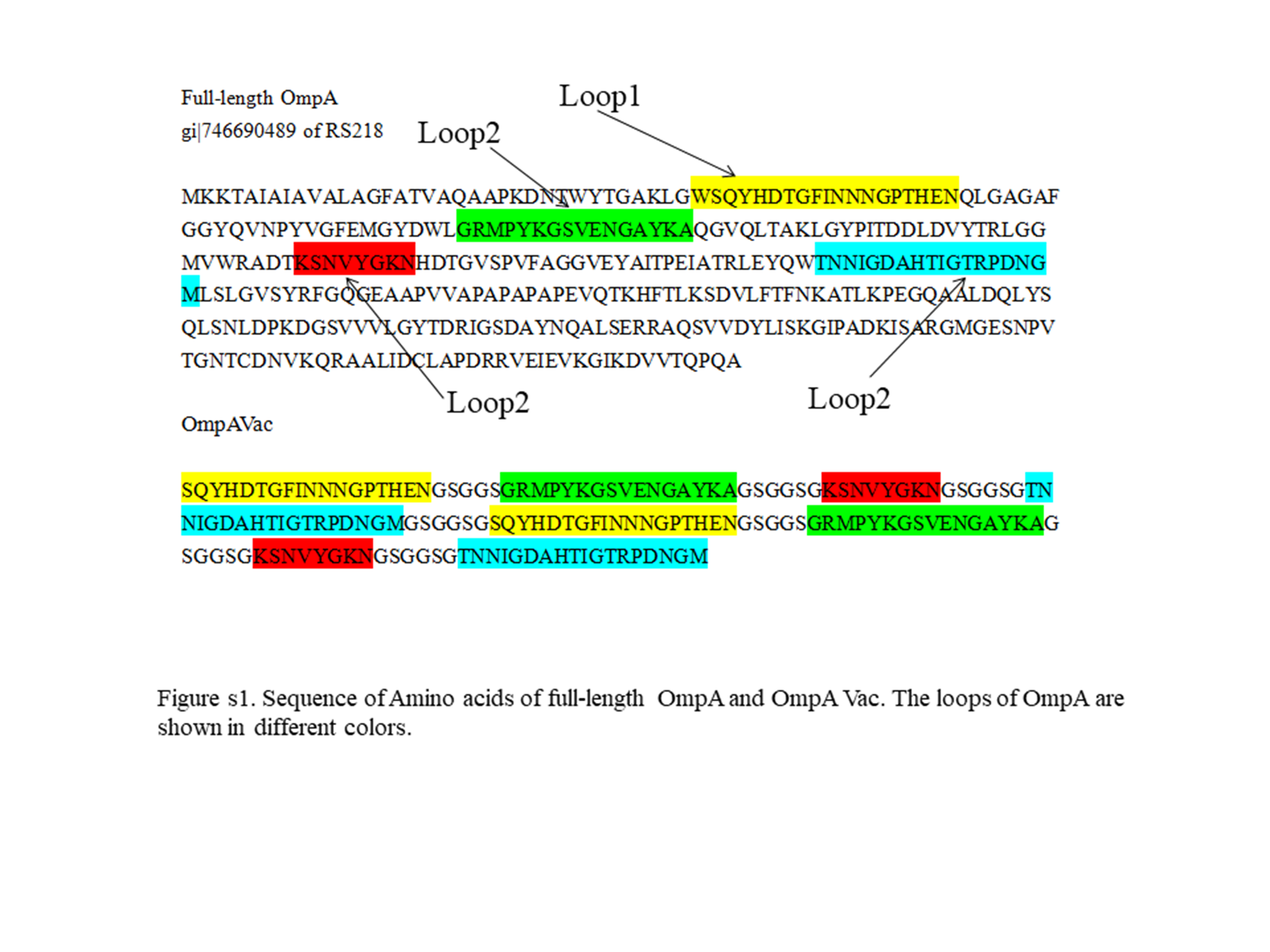

Supplement: Supplementary file 2 [file Image_1.TIF]
